# Glycaemic, blood pressure and cholesterol control in 25 629 diabetics

**DOI:** 10.5830/CVJA-2015-050

**Published:** 2015

**Authors:** Y Pinchevsky, N Butkow, T Chirwa, FJ Raal

**Affiliations:** Department of Pharmacy and Pharmacology, School of Therapeutic Sciences, Faculty of Health Sciences, University of the Witwatersrand, Johannesburg, South Africa; Department of Pharmacy and Pharmacology, School of Therapeutic Sciences, Faculty of Health Sciences, University of the Witwatersrand, Johannesburg, South Africa; Division of Epidemiology and Biostatistics, School of Public Health, Faculty of Health Sciences, University of the Witwatersrand, Johannesburg, South Africa; Carbohydrate and Lipid Metabolism Research Unit, Division of Endocrinology and Metabolism, Faculty of Health Sciences, University of the Witwatersrand, Johannesburg, South Africa

**Keywords:** type 2 diabetes mellitus, guidelines, goal achievement

## Abstract

**Objective:**

To examine and compare the extent to which people with type 2 diabetes (T2DM) are achieving haemoglobin A_1c_ (HbA_1c_), blood pressure (BP) and LDL cholesterol (LDL-C) treatment targets.

**Methods:**

A review of databases (MEDLINE Ovid, Pubmed and Sabinet) was performed and limited to the following terms: type 2 diabetes mellitus AND guideline AND goal achievement for the years 2009 to 2014 (five years).

**Results:**

A total of 14 studies (25 629 patients) were selected across 19 different countries. An HbA_1c_ level of 7.0% (or less) was achieved by 44.5% of subjects (range 19.2–70.5%), while 35.2% (range 7.4–66.3%) achieved BP of 130/80 mmHg (or less), and 51.4% (range 20.0–82.9%) had an LDL-C level of either 2.5 or 2.6 mmol/l (100 mg/dl or less).

**Conclusion:**

Despite guideline recommendations that lowering of HbA_1c_, BP and lipids to target levels in T2DM will lead to a reduction in morbidity and mortality rates, we found that control of these risk factors remains suboptimal, even across different settings.

## Objective

Diabetes mellitus (DM) is a chronic, progressive condition leading to significant morbidity and premature death, and is an economic burden to any healthcare system. According to the International Diabetes Federation (IDF), there were 366 million people living with diabetes in 2011.^1^ By 2035, it is predicted that more than half a billion people will have the disease.

Trends in urbanisation and the adoption of unhealthy Western lifestyles have begun to affect low- and middle-income countries (LMICs). A prime example of this is South Africa, which previously had the dubious pleasure of infectious diseases being the primary source of mortality. Today, expansion of non-communicable diseases (NCD) is beginning to manifest and deplete the already strained health resources available.^2^

Rather than being limited to glycaemia alone, the management of type 2 diabetes mellitus (T2DM) includes multiple priorities, including identification and treatment of other modifiable risk factors. It is widely accepted that T2DM is associated with cardiovascular disease (CVD) and increased mortality rates.^3^

In addition to lifestyle changes, the importance of reduction in levels of low-density lipoprotein cholesterol (LDL-C) and blood pressure (BP) has become an essential primary goal for the prevention of CVD in T2DM.^4^,^5^ Furthermore, improved outcomes of diabetes-related chronic microvascular complications (retinopathy, neuropathy and nephropathy) are achieved through substantial reductions in incidence of both hyperglycaemia and hypertension. It is on the basis of this research that the Society for Endocrinology, Metabolism and Diabetes of South Africa (SEMDSA) recommends that most adults with diabetes should aim for an HbA^1c^ level of 7.0%, BP of 140/80 mmHg and LDL-C level of 2.5 mmol/l or less.^6^

There are many gaps in the management of T2DM that are proving difficult to close. Studies have revealed how clinical practice differs from clinical trials in that T2DM patients often cannot reach guideline-recommended targets. One of the ways to improve clinical outcomes is by comparing the performance of one clinical setting against another. In this study, our aim was to compare the achievement of the critical quality indicators: glycaemic, BP and lipid control in T2DM patients from different countries worldwide, in an attempt to benchmark which approach has been most successful.

## Methods

This study was a literature review using Ovid MEDLINE, Pubmed and Sabinet databases. Studies included were those conducted in the past five years (2009–2014) and limited to the following key terms: type 2 diabetes mellitus AND guideline AND goal achievement (HbA_1c_, glycated haemoglobin, blood pressure, systolic, diastolic, lipids, cholesterol, LDL cholesterol). We also reviewed a selected number of reference lists of other reviews and hand-searched several medical journals.

Studies that reported achievement of guideline-recommended targets of major risk factors for T2DM were included. The primary objective of this review was to provide an overview of achievement of major risk-factor targets (HbA_1c_, BP and LDL-C) in the treatment of a sample of T2DM patients from different parts of the world. Specifically, the objectives would be addressed through comparison of the achievement of HbA_1c_, BP and LDL-C targets, according to local or international guidelines, across different study samples.

The following data were extracted from the studies: author details, year of publication, study location, cohort size and achievement of major risk factors (combined systolic and diastolic BP, and HbA^1c^ and LDL-C levels). As different samples of study countries followed different guideline targets, flexibilities around these differences was needed. Studies selected for this article may have differed in the following parameters: recruitment and randomisation methods, total number of study participants recruited, study sites (e.g. single or multicentre), gender ratios, ethnicity ratios, timelines of results presented (e.g. single or longitudinal data) and periods of enrollment.

To compare results, we standardised (or converted or conformed) certain measurement units in order to maintain consistency (e.g. LDL-C in mmol/l instead of mg/dl). The control or baseline results of studies were reported instead of interventional group data. Only the latest data were selected from studies with multiple time periods.

Studies excluded from the review had one or more of the following characteristics: non-English language, studies conducted before 2009, participating patients younger than 18 years of age, participants reported to have had any diabetes other than T2DM (e.g. gestational, type 1 or steroid induced), studies that reported insufficient data or less than two of the three major risk factors being compared, and studies that consisted of large HMO claims databases. The latter was chosen as an exclusion criterion as larger-sized cohort studies would have biased the results of this review

Data presented in this article were collected from the results of other studies and are limited to the authors’ definitions of control. This review did not allow for the access of patient-level data of different studies included in the review to be accessed. It was assumed that all data extracted for this study were collected from the medical records of patients who willingly participated in the studies included in this review. The relevant data were captured into a secure database using Microsoft Excel 2010. Ethical approval was obtained from the University of the Witwatersrand Human Research Ethics Committee (Medical).

## Results

The authors of this study set out to determine how diabetes care compared across different settings, given the healthcare challenges faced especially by under-resourced areas. Of the 511 (154 from Ovid MEDLINE + 32 Pubmed + 325 Sabinet) titles initially identified between 2009 and 2014, 14 studies fulfilled the inclusion criteria. These 14 studies originated from 19 different countries (some studies included more than a single country) and we enrolled a total of 25 629 patients.

There were 17 high-income, one upper-middle- (South Africa) and one low-income (Uganda) country included in the review (grouped according to the United Nations’ economies by per-capita country classification).^7^ Cohort sizes ranged from 50 to 4 926 patients. Twelve studies contained results for all major risk factors (HbA_1c_, BP and LDL-C), while the rest included at least two-thirds of the measured risk factors. There were eight studies (57.1%) that defined treatment targets as per the American Diabetes Association.^8^ The characteristics of each study are outlined in Table 1.

**Table 1:** Study characteristics

				*Achievement of target*		
*First author (reference)*	*Year of publication*	*Location*	*Cohort size (n)*	*HbA_1c_ (< 7%)*	*BP (< 130/ 80 mmHg)*	*LDL-C (< 2.6mmol/l)*
Al-Taweel^9^	2013	Kuwait	652	19.2	46.0	-
Braga^10^	2012	Canada	3002	52.6	53.6	64.2^||^
Casagrande^11^	2013	USA	4926	52.5	51.1	56.2
Goderis^12^	2009	Belgium	2495	54.0	50.0^‡^	42
Hermans^13^	2013	Belgium, Greece, Luxembourg, Portugal, Spain, UK	3996	49.2	27.3^‡^	40.8
Kibirige^14^	2014	Uganda	250	20.8	56.0^§^	20.0
Klisiewicz^15^	2009	South Africa	150	30.7	21.3^‡^	50.7^||^
Lee^16^	2009	Korea	926	49.2	66.3	51.0
Morren^21^	2010	Trinidad	225	56.6^†^	53.6	49.3**
Pinchevsky^17^	2013	South Africa	666	26.2	45.8	53.8^||^
Sease^18^	2013	USA	95	35.8	62.1	82.9
Stone^19^	2013	Belgium	1044	59.7	27.6	49.7
Stone^19^	2013	France	1056	65.3	14.9	52.4
Stone^19^	2013	Germany	959	48.6	7.4	30.7
Stone^19^	2013	Ireland	950	53.4	24.9	76.9
Stone^19^	2013	Italy	984	35.7	20.8	40.4
Stone^19^	2013	Netherlands	1021	70.5	20.3	58.9
Stone^19^	2013	Sweden	550	56.5	27.1	47.3
Stone^19^	2013	UK	1033	39.1	25.0	74.5
Umar-Kamara^22^	2011	USA	50	60.0*	24.0^¶^	-
Webb^20^	2014	South Africa	599	27.0	32.0^#^	33.0^||^

Exceptions to the above targets are indicated by the following: ^†^HbA1c < 6.5%;*HbA1c < 8.0%; ^‡^systolic blood pressure only < 130 mmHg; ^§^systolic/diastolic blood pressure < 140/80 mmHg; ^¶^systolic/diastolic blood pressure < 140/90 mmHg; ^||^low-density lipoprotein cholesterol < 2.5 mmol/l; **total cholesterol < 5.18 mmol/l.

In 12 studies (25 354 patients) that used an HbA_1c_ level of 7.0% or less to define control, 44.5% (range 19.2–70.5%) of patients achieved target.^9^-^20^ In two studies (275 patients) where HbA_1c_ level was defined as < 6.5 and < 8.0%, respectively, 56.6 and 60.0% of patients reached their targets, respectively.^21^,^22^

In eight studies (18 089 patients), which had the definition of target BP of 130/80 mmHg or less (systolic and diastolic combined), 35.2% (range 7.4–66.3%) of patients achieved target.^9^-^11^,^16^-^19^,^21^ In four studies (7 240 patients) where systolic BP targets of 130 mmHg or less (alone) defined control, 32.7% (range 21.3–50.0%) of the subjects achieved target.^12^,^13^,^15^,^20^ In two studies (300 patients) with a BP target of either < 140/90 or < 140/80 mmHg, 24.0 and 56.0% of patients achieved goal, respectively.^14^,^22^

In the 11 studies (24 702 patients) that used LDL-C levels of either 2.5 or 2.6 mmol/l (100 mg/dl or less) to define control, 51.4% (range 20.0–82.9%) of patients achieved goal.^10^-^20^ One study (225 patients) with a total cholesterol target of < 200 mg/ dl (5 mmol/l) had 49.3% of patients at goal.^21^ Two studies (702 patients) did not measure lipid levels.^9^,^22^

In general, more patients reached target for LDL-C than for HbA_1c_ levels, with the poorest achievement of targets being BP. The widest variability of target achievement was LDL-C (variation of 62.9%), followed by BP and then HbA_1c_ (least variability). The highest and lowest achieved targets were those by an American (LDL-C, 82.9%) and a German study (BP, 7.4%), respectively.^18^,^19^

## Discussion

The quality of diabetes care cannot simply be measured across proportions of patients achieving guideline targets. However, a broad overview of the quality of care can be gauged when comparing target adherence across different countries, especially with adequately sized samples of patients. Hence the reason for this review, where countries from various economies were compared, according to achievement of modifiable risk factors against the guidelines. Based on the results of other studies, this review set out to establish the achievement of major risk-factor targets (HbA_1c_, BP and LDL-C levels) in the treatment of DM patients in different parts of the world.

Given the increasing prevalence of T2DM, effective management of critical diabetes risk factors can significantly contribute towards improved outcomes. Attaining targets requires improved methods to increase adherence to lifestyle (exercise/diet) and pharmacological interventions. From this review, it was evident that certain studies appeared to be more successful at managing patients’ risk factors than others.

Practitioners achieving better guideline adherence should be encouraged to share their management strategies for implementation with other healthcare facilities. Hermans et al. found that by benchmarking the level of care of ‘three paramount cardiovascular risk factors’ in a primary care setting has in itself led to a clinically significant improvement in T2DM care over time.13 There is also evidence to suggest that performance with regard to management of a disease, when compared between a physician and his/her colleagues, has brought about an intellectual, emotional and competitive incentive for change.^23^

The most critical ways of reducing T2DM complications is by collectively managing HbA_1c_, BP and LDL-C levels. More patients achieved LDL-C than HbA_1c_ targets in the studies reviewed, potentially owing to the progressive nature of the disease, where β-cell function gradually declines over time. BP control was the least-achieved risk factor across all the studies, and according to McLean *et al.*, may have occurred due to the ‘inadvertent under emphasis’ of treating T2DM-associated risk factors (such as hypertension, when there is strong emphasis on glucose control).^24^ Perhaps it was due to inadequate dosages, poor adherence to medication, poor access to follow-up care or a combination of these. A well-designed, randomised, controlled trial may help address these questions.

Once considered rare in sub-Saharan Africa, the prevalence of T2DM is rapidly increasing. As many as four out of every five diabetics reside in LMICs, many of whom remain undiagnosed.^1^ T2DM is a complex, resource-intensive disease requiring multifactorial yet individually tailoured, lifelong treatment.

Most of the studies found and included in this review were from higher-income countries. However patterns of poor control rates were common across all settings. For instance, less than 40% of patients from the USA, Europe (specifically Italy) and the UK studies (all high-income countries) achieved HbA_1c_ levels (< 7%) comparable with those of lower- to upper-middleincome countries (Uganda and South Africa, respectively).^14^,^17^-^20^ Similarly, the combined results of six European countries, and other individual studies, had less than half of patients at LDL-C target, as seen in two separate non-high-income countries.^13^,^14^,^20^ Yet on the other hand, and possibly as expected from moredeveloped nations, two to three times more patients from separate European (specifically the Netherlands) and a USA study achieved HbA_1c_ (< 7%) and LDL-C (< 2.6mmol/l) targets in comparison with a lower-income country, respectively.^14^,^19^,^22^

The differences across the sites in their abilities to achieve guideline targets may be attributed to socio-economic reasons. In resource-rich settings, where patients supposedly receive the extra time required for diabetes care through more regular physician interactions or appointments, appropriate reminder systems and adherence monitoring, this may improve the standards of diabetes care received. Lower-income countries face the realities of inadequate healthcare infrastructure, regular medication stock outs, few educational programmes and minimal healthcare facilities/professionals.^25^ This literature review covered the influences of multiple background factors occurring across healthcare systems in different countries, hence the differences in targets achieved across the environments studied.

As described above, Africa faces many healthcare challenges, both within and between countries. Despite resource constraints, by targeting the modifiable risk factors associated with DM, there is still the potential for improvement, and better patient outcomes. This review serves to highlight the proportion of patients achieving guideline targets across different settings. The aim of this review was to serve as a benchmark for those countries selected, in order to measure their performance against each other in terms of achieving guideline targets.

By recognising those healthcare settings with increased patient numbers achieving guideline targets, this could allow for future studies to identify the mechanisms and processes used to achieve their targets. Areas of interest for the improvement of diabetes care could include: organisational characteristics such as improved implementation of adherence to clinical guidelines (evidence-based), identification of individuals to act as guideline champions to deliver more performance measures, and feedback to healthcare providers on progress made. Perhaps, once identified, the settings achieving less-favourable control of modifiable risk factors may begin to explore approaches used in the more successful settings. In addition, given the chronic progressive nature of DM, it is hoped that attention will be prioritised not just on treatment but also on prevention strategies in those settings wishing to improve their level of diabetes care offered.

It has been predicted that the ageing populations of LMICs will face a significant increase in mortality rate due to NCDs over the next 25 years.26 Although not included in this review, a previous South African study revealed that only 30.4% of the 899 patients achieved HbA_1c_ levels < 7%, which is similar to the three studies included in this review from the same locale.27 The three South African studies included in this review had noticeably fewer patients at HbA_1c_ goal in comparison with other countries. One of the reasons for this may have been that all the South African studies included in this review were from the public sector, which has often been described as ‘overburdened’, and due to resource constraints, cannot always offer appropriate levels of healthcare or access to the most modern diabetes treatments currently available in South Africa’s private sector or in those high-income countries included in this study. Furthermore, many of the patients serviced in South Africa’s public sector settings originate from a lower socioeconomic background, which may indicate lower educational levels or limited access to healthier lifestyle choices. Perhaps HbA_1c_ level is still the most challenging of risk factors to control, especially in less-developed economies.

## Study limitations

Studies included in this review differed with regard to guidelines or targets, however, we tried to overcome this by consistently capturing and comparing similar risk factors using standardised units. Patients selected for the studies included in the review were treated to ‘moving targets’ and therefore studies conducted in the past five years only (2009–2014) were included, as recent guidelines have more similar targets.

Some eight out of the 14 studies provided information on the type of methods used for clinical and laboratory-based measurements, but with differing levels of detail. Notably, only two studies confirmed use of DCCT-standardised laboratory analysers for HbA_1c_ analysis. By converting to similar units (e.g. LDL-C from mg/dl to mmol/l), target values in this retrospective study (Table 1) were presented in a format that would allow for comparison. Ideally, a centralised laboratory should have been used for measurements included in this study, however, we relied on previously obtained measurements from other studies. We therefore cannot guarantee the accuracy or precision of measurements in the studies selected for this review, as methodologies may have differed. Studies from resourcerich settings may have implemented newer, more sophisticated and improved methods for A_1c_ and LDL-C measurements, influencing the results of those specific studies.

This review did not stratify the selected individual studies according to patient profiles, severity of disease, clinical settings (clinic or hospital) or involvement of specialists (factors affecting how individuals are managed and able to reach guideline targets). Although smoking is considered a critical risk factor in the prevention and management of CVD, it was not included as a crucial study parameter for this review (partly due to many studies not reporting this).

Although previously identified as a source of bias, we only included studies published in English, which according to an analysis, has little effect on summaries of treatment effect estimates.^28^ Publication bias may have occurred in our study in that a single reviewer (author) carried out the searches without the use of specific methodology (e.g. Cochrane data system).

## Conclusion

The results presented in this study demonstrate that T2DM patients remain inadequately controlled for their cardiovascular risk factors. Our review revealed that control of major risk factors did not differ significantly between countries or healthcare settings. There is substantial room for improvement in the way T2DM patients are being managed for their condition. Further efforts through multidisciplinary action to improve guideline adherence is critical for the prevention or delay of diabetesrelated complications.
